# A prospective study of two isothermal amplification assays compared with real-time PCR, CCNA and toxigenic culture for the diagnosis of *Clostridium difficile* infection

**DOI:** 10.1186/s12866-016-0635-5

**Published:** 2016-02-12

**Authors:** Martina Neuendorf, Raquel Guadarrama-Gonzalez, Birgit Lamik, Colin R. MacKenzie

**Affiliations:** Institute of Medical Microbiology and Hospital Hygiene, University Hospital, Heinrich-Heine University, Düsseldorf, Germany

**Keywords:** Clostridium difficile, Molecular diagnosis, Isothermal amplification, Real-time PCR, CDI

## Abstract

**Background:**

New molecular methods of detecting *Clostridium difficile* infection (CDI) provide the routine lab with a sensitive random access method to produce results that are available in a shorter time than traditional methods.

**Methods:**

In this prospective study a total of 989 stool specimens were tested over a period of 16 months in parallel using two isothermal amplification assays, AmpliVue® (Quidel) and Illumigene® (Meridian) and the results compared to those from toxigenic culture. In addition all specimens were tested using a cytotoxic cell neutralisation assay (CCNA) and three different Real-time PCR targeting a *C. difficile*-specific 16S rDNA sequence or the toxin genes *tcdA*, *tcdB/tcdB027* or *cdtB*.

**Results:**

AmpliVue® was positive in 242 (24.5 %) and Illumigene® in 228 (23.1 %) specimens. 167 (16.9 %) specimens were positive in toxigenic culture. Real-time-*tcdA* and -*tcdB* PCR was positive in 211 (21.3 %) specimens, Real-time*-cdtB* PCR was positive in 101 (10.2 %) specimens and *C. difficile*-PCR (16S rDNA) in 267 (27.0 %) specimens.

**Conclusions:**

The respective sensitivity, specificity, positive predictive value and negative predictive value compared to toxigenic culture were 91, 89, 62 and 98 % for AmpliVue® and 91, 91, 67 and 98 % for Illumigene®.

## Background

*Clostridium difficile* infection (CDI) is the leading cause of health-care associated diarrhoea most frequently associated with antecedent antibiotic therapy. The incidence of CDI in Europe is rising steadily and has considerable influence on patient morbidity and mortality [1]. The clinical presentation varies widely from mild, watery diarrhoea to pseudomembranous colitis, toxic megacolon and sepsis [2] and in addition recurrent CDI has become a major problem possibly related to binary toxins as virulence factors [3, 4].

Rapid diagnosis forms a cornerstone for patient management and early isolation, and adequate (antibiotic) therapy of infected patients may reduce transmission and disease severity [5, 6]. The laboratory detection of *Clostridium difficile* is increasingly focused on toxin detection as routine toxigenic culture has a long turnaround time [7]. A *C. difficile*-specific glutamate dehydrogenase antigen detection assay can be used as a sensitive screening test but has a low specificity and thus requires confirmation, usually by detection of one or both toxin genes [8] or antigen detection using an EIA, which has a low sensitivity. Isothermal amplification assays are easy to use, have low hands on and turnaround times and are highly sensitive [9–12]. European and American guidelines have recommended a two-step testing algorithm for the laboratory diagnosis of CDI consisting of a sensitive antigen test followed by a more specific confirmatory test [13, 14] although more recent guidelines have recommended the use of NAATs as stand-alone tests [15]. Due to insufficient data isothermal amplification assays have not generally been included in the recommendations concerning NAAT [15]. A recent meta-analysis of published data for loop-mediated isothermal amplification shows the accuracy of this type of isothermal amplification method [9]. The optimal combination of tests is largely dependent on the local conditions and resources. In this large prospective study we compared the performance of two different isothermal amplification assays (AmpliVue®, helicase dependant amplification and Illumigene®, loop-mediated isothermal amplification) with toxigenic culture and additionally with an in-house Real-time PCR and cytotoxic cell neutralisation assay in a routine diagnostic laboratory setting.

## Methods

From January 2013 to April 2014 consecutive liquid stool specimens received by the laboratory with a request for *C. difficile*-toxin assay from hospital wards and out-patient departments of three tertiary care hospitals were included in the study and fully evaluated. The persons performing the tests were blinded to the results of the other tests. The study was approved by the ethics committee of the medical faculty of the Heinrich-Heine University of Düsseldorf. Specimens were tested by two commercial isothermal amplification assays, toxigenic culture (TC), cytotoxic cell neutralisation assay (CCNA) and by Real-time PCR (Real-time-*tcd* PCR) targeting gene sequences for toxin A, B, and B027 (a sequence incorporating a mutation found in the *tcdB* gene in ribotype 027 strains), binary toxin B (Real-time-*cdtB* PCR) and a *C. difficile-*specific 16S fragment (Real-time-16S PCR). In previous work we found no *C. difficile* strains with discordant *cdtA* and *cdtB* results and thus the *cdtB* gene was used as a marker for the presence of both binary toxin genes (unpublished data). Sequencing of the *slpA* gene was performed on all available *C. difficile* isolates. The reference method was toxigenic culture.

All specimens were tested according to the manufacturers’ instructions within 48 h after delivery to the laboratory. If necessary the specimens were stored at 4 °C until testing was performed. In brief, the Illumigene® assay employs loop-mediated isothermal DNA amplification (LAMP) to detect a gene segment of the *tcdA* gene in the pathogenicity locus (PaLoc) present in all known toxigenic *C. difficile* strains. The AmpliVue® assay utilizes helicase-dependant amplification (HDA) for the detection of a conserved sequence of the *tcdA* gene. Positive and negative controls for both assays were provided by the manufacturers and were performed weekly. All stool specimens were cultured for *C. difficile* by inoculating a portion of the stool sample onto *Clostridium difficile* selective agar (bioMérieux, Germany) and incubating under anaerobic conditions for 48 h. Growth of colonies suspected to be *C. difficile* were identified by MALDI-ToF (Vitek® MS, bioMérieux, Germany). *C. difficile* isolates were stored in glycerine stocks at −80 °C and re-cultured on blood agar for *slpA* sequencing.

CCNA was performed by re-suspending approximately 100 μl of fresh stool in 500 μl normal saline and centrifuging at 850 g for 1 min. Thereafter the supernatant was passed through a 0.2 μm filter and 50 μL of two dilutions, 1:5 and 1:50 in sterile normal saline, added in duplicate to four wells containing a monolayer of BGM (Buffalo Green Monkey) cells (Sigma-Aldrich, Munich). To one well of each dilution polyclonal anti-toxin antibody was added (Techlab, Blacksberg, USA). A cytopathic effect only in the well without anti-toxin was interpreted as a positive result. Toxigenic culture was performed by adding *C. difficile* culture supernatant to the CCNA instead of stool and results were interpreted in a similar procedure.

DNA extraction from stool specimens was performed by mixing approximately 10 μl of the stool specimen in 500 μl PBS and centrifuging at 850 g for 1 min. A 200 μl aliquot of the supernatant was extracted using the DNA tissue kit and EZ1 BioRobot (Qiagen, Germany) and eluted in 100 μl. The eluates were kept at −20 °C until tested.

For *slpA* sequencing 2.5 μl DNA eluate was added to 1 μl Peqgold Hot-Start mastermix (Peqlab, Erlangen), 20.5 μl distilled water and 1 μl (3 μM) primers *slpA* 19 and *slpA* 22 (Table 1). The PCR was performed on an Eppendorf Mastercycler pro thermocycler (Wesseling-Berzdorf, Germany) as follows: 95 °C for 5 min and subsequently 35 cycles comprising 95 °C for 20 s and 55 °C for 180 s, then incubation at 74 °C for 30 s. A final step at 74 °C for 5 min completed the PCR. The amplified DNA was sequenced by the Sanger method in the university molecular biology core facility (BMFZ, Biologisch-Medizinisches Forschungzentrum).

**Table 1:** Primers and probes

Gene	Primers/Probes	PCR-product (bp)	Acc-number	Gene region (bp)
tcdA	tcdA F: 5-´ATTCCAATACAAGYCCTGTAGAAAA-3´	85	AM180355	796102–796186
	tcdA R: 5- TTTATGTATTCAAGARCAATATCACTGACT-3´			
	tcdA-S: 5´Fam-RATTTACATTTTGTATGGATAGGTGGAG-BHQ-1-3´			
tcdB	tcdBF: 5´-AACAGGTGTATTTAGTACAGAAGATGGATT-3´	85	AM180355	793077–793161
	tcdBR: 5´-AATTGCTTCTCCTTCTAGGTTTTCAT-3´			
	tcdB-S: 5´-Hex-AAATATTTTGCCCCAGCTAATACACTT-BHQ-1-3´			
tcdB027^a^	tcdBF: 5´-AACAGGTGTATTTAGTACAGAAGATGGATT-3´	85	FN665654	708928–709012
	027tcdBR*: 5´-AATTGCTTC**C**CC**C**TCTAG**A**TTTTCAT-3´			
	027tcdB-S:5´-Hex-AAATATTTTG**CT**CCAGC**AG**ATACACTT-BHQ-1-3´			
16S	CdF: 5´-TGTACACACGGATAACATACCGAAA-3´	131	AM180355	125189–125289
	CdR:5´-CCGTTACCTTACCAACTAGCTAATCA-3´			
	CdS5´-Fam-CATCTCTTGAATATCAAAGGTGAGCCAGTACAGG-BHQ-1-3´			
cdtB	cdtBfor: 5´GATGATCCATTTATCCCAAATAACAA-3´	132	FN665654	2833044–2833175
	cdtBrev:5´GTCCTTAATAGTATATCCATTTCGTTCATATG-3´			
	cdtBS:5´Hex-TTCTTTGACCCAAAGTTGATGTCTGATTGGG-BHQ-1-3´			
slpA (Kato et al. 2004)	slpAcom22 : 5'-GCWGTYTCTATTCTATCDTYWCC-3'	23	AM180355	3253526–3254737
	slpAcom19 : 5'-GTTGGGAGGAATTTAAGRAAtG-3'	22		

^a^Bolded bases represent a difference from the standard *tcdB* primer or probe

For all Real-time PCRs the mastermix per specimen was composed of 12.5 μl Qiagen Mastermix and 2.5 μl (3 μM) each of forward and reverse primers (Table 1), 2.5 μl probe and 2.5 μl H_2_O. 2.5 μl from the DNA-eluate were added to the mastermix and heated at 50 °C for 10 min and then 95 °C for 10 min and thereafter placed in a thermocycler (BioRad, München) for 44 cycles comprising 95 °C for 15 s and 60 °C for 60 s.

The sequences for the primers and probes for the Real-time PCR and *slpA* analysis are shown in Table 1.

## Results

A total of 989 stool specimens from 828 patients were included in the study. The number of positive specimens in toxigenic culture was 167 (17 %). The AmpliVue® assay was positive in 242 (25 %) specimens and Illumigene® assay in 228 (23 %) specimens. Real-time-*tcd* PCR was positive in 211 (21 %) specimens (of these 79 (8 %) were positive for the *tcdB027* gene) and *C. difficile*-16S-PCR was positive in 267 (27 %) specimens.

Isothermal amplification assay results discrepant to toxigenic culture are shown in Tables 2 and 3. A total of 90 (9 %) specimens tested with AmpliVue® and 75 (8 %) specimens tested with Illumigene® were positive by isothermal amplification but negative in the toxigenic culture (i. e. false positive). Sixty three of these specimens were positive in both isothermal amplification methods. On the other hand 15 (2 %) specimens tested with AmpliVue® and 14 (1 %) specimens tested with Illumigene® were negative in isothermal amplification but positive in the toxigenic culture (i. e. false negative). Of these 12 specimens were negative in both isothermal amplification assays. *C. difficile* strains in which no toxin production was detected in bacterial culture supernatants were classified non-toxigenic strains. 20 of 34 stool specimens containing non-toxigenic strains were positive in the AmpliVue® assay and 19 strains were positive in the Illumigene® assay. The respective sensitivity, specificity, positive predictive value (PPV) and negative predictive value (NPV) for the isothermal amplification assays compared to toxigenic culture were 91, 89, 62 and 98 % for AmpliVue® and 91, 91, 67 and 98 % for Illumigene®. The sensitivity, specificity, PPV and NPV for Real-time *tcd* PCR compared to toxigenic culture were 91, 93, 73 and 98 % respectively.

**Table 2:** Discrepant results for isothermal amplification: false positive specimens

N	AmpliVue®	Illumigene®	Culture	TC	Real-time-*tcd* PCR	16S PCR
17	+	+	+	-	+	+
1	+	+	+	-	-	+
30	+	+	-	-	+	+
4	+	+	-	-	-	+
11	+	+	-	-	-	-
1	+	-	+	-	-	+
1	+	-	-	-	+	+
3	+	-	-	-	-	+
18	+	-	-	-	-	-
1	-	+	+	-	+	+
2	-	+	-	-	+	+
8	-	+	-	-	-	-
1	+	invalid	+	-	+	+
1	+	invalid	-	-	-	+
1	+	invalid	-	-	+	+
1	+	invalid	-	-	-	-
1	Invalid	+	-	-	-	-

TC, toxigenic culture

**Table 3:** Discrepant data for isothermal amplification: false negative specimens

N	AmpliVue®	Illumigene®	TC	Real-time-*tcd* PCR	Real-time 16S PCR
1	-	-	+	+	+
2	-	-	+	-	+
9	-	-	+	-	-
2	-	+	+	-	+
1	-	+	+	+	+
2	+	-	+	+	+

TC, toxigenic culture

**Fig. 1:**
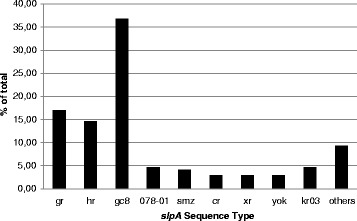
Prevalence of slpA types in the study isolates. All cultures were subjected to typing by determining the slpA sequence as described in the methods

For general epidemiological purposes and to determine if there was any correlation between strain type and the results of isothermal amplification or toxigenic culture all cultures were typed by *slp*A-sequencing. Of the *C. difficile* strains 171 were typable by *slpA* sequencing the distribution of which is shown in Fig. 1. Three *slpA* types made up the majority (69 %) of the strains: the most common *slpA* type was gc8 (63 specimens, 37 %) (associated with ribotype 027 [16]); followed by gr (29 specimens, 17 %; 27 of which were gr-01 and 2 were gr-04) and hr (25 specimens, 15 %; 19 of which were hr-01, 4 were hr-02, 1 was hr-05 and 1 was hr-06). 8 (5 %) specimens were typed as 078–01 or kr03. All other *slpA* types were represented by less than 8 specimens each. 14 specimens of the 171 specimens analysed by *slpA* sequencing were atoxigenic as they were neither positive in TC nor in Real-time-*tcd* PCR. Atoxigenic *slpA* types were xr and 078 (3 specimens each) and one strain each was nc, cr, ac, fr. Of the 79 specimens positive in the *tcdB*027 Real-time PCR 61 specimens were available for *slpA*-typing. 57 (93 %) of these specimens were type gc8 and of 63 gc8 types 57 (90 %) were *tcdB*027-positive.

The binary toxin gene (*cdtB*-PCR) was detected in 87 specimens. A correlation between Real-time *tcdB*027 PCR and *cdtB* PCR positivity was high (70 of the 87 binary toxin positive specimens (80 %) were Real-time-*tcdB*027 PCR positive).

## Discussion

To our knowledge this is the largest reported study comparing the isothermal amplification assays from AmpliVue® and Illumigene® with a Real-time PCR in reference to toxigenic culture. A study reported by Deak et al. directly comparing the AmpliVue® assay and a Real-time PCR assay by Simplexa® (Simplexa ^TM^, Focus Diagnostics) with the Illumigene® assay in a sample size of 200 found a sensitivity and specificity of 96 % and 100 %. Other recent studies with a lower number of specimens using TC as reference method have also revealed high sensitivities and specificities for AmpliVue® and Illumigene® [10–12, 17, 18].

In general molecular tests have many advantages: They are easy to perform and often associated with high sensitivity and specificity. Nevertheless there are some disadvantages. Firstly, the assays are performed in stool containing a large amount of extraneous DNA which may interfere with the detection [11]. Recent studies however have not shown much cross reactivity [19, 20]. Secondly, the mere presence of toxin genes is not proof of toxin expression. Therefore only patients with an appropriate clinical suspicion of CDI should be tested by the laboratory [11, 21] since the carriage of *C. difficile*, even toxigenic strains, is not necessarily diagnostic of disease or in itself an indication for therapy [22, 23]. In settings with a low prevalence of CDI the low positive predictive value of the isothermal tests as shown in this study may lead to a rather high number of laboratory diagnoses of CDI, in which perhaps another cause for the diarrhoea is present. This is especially important in clinically complicated cases, in which multiple factors may be relevant.

The relatively low PPV in our study compared to that of Hong et al. may possibly be due to a lower sensitivity of the reference method in our study. The number of specimens positive in 16S-PCR (106) that were *C. difficile* culture-negative would support this hypothesis, however the detection of DNA from *C. difficile* alone does not necessarily relate to the presence of viable bacteria and since the CCNA was negative in all these cases it would seem probable that if indeed bacteria were present and viable, they were not producing significant amounts of toxin and therefore not causing CDI. This raises the important question as to the clinical usefulness of such highly sensitive methods to detect CDI and if NAAT methods may in fact not overcall CDI. A contrary view is that since CDI is thought to be essentially underdiagnosed [24, 25], the high sensitivity of isothermal amplification assays may contribute to a better detection of disease.

In 34 (3.4 %) specimens the toxigenic culture was negative despite a positive culture for *C. difficile* indicating non toxin-producing strains. In comparison the Real-time-*tcd* PCR was positive in 56 cases, in which toxigenic culture was negative resulting in a specificity and PPV of 93 and 73 %. According to Buchan et al. false positive results can occur due to the presence of non-viable bacteria or residual DNA or because the isothermal amplification tests have a lower level of detection than toxigenic culture (i. e. true positive). As a control in their study they used another PCR targeting a different region of the *C. difficile* chromosome than that targeted by the isothermal amplification assay, the results of which confirmed the results of the latter. This would support the purported higher sensitivity of the isothermal amplification assay [26]. Following this argument the performance of the isothermal amplification assays in terms of true specificity and PPV would be better if the reference method in our study was more sensitive.

In the present study we found only one minor difference between the two isothermal assays: A higher number of invalid results (26 specimens) were obtained using the Illumigene® assay; whereas only 11 specimens were invalid using the AmpliVue® assay. The relatively high number of non-evaluable tests using Illumigene® was due to sample overloading at the beginning of the study period, which improved with experience and in the latter half of the study was negligible. Furthermore Illumigene® invalid results are known to be caused by blood-containing samples [27, 28]. In this study very few patients presented with bloody diarrhoea (data not shown). However we were able to confirm this in 2 macroscopically bloody samples. This would be a disadvantage of the assay for patients with severe CDI and bloody stools unless an additional DNA-purification step is added prior to testing.

To evaluate the practicality of the isothermal amplification assays the number of steps, hands on time and turnaround time were determined. Both are equivalent in terms of practicality in the routine laboratory. Each assay had 6 (Illumigene®) and 7 (AmpliVue®) steps with hands on time for 10 specimens of 14 min (Illumigene®) and 12 min (AmpliVue®) and total turnaround time was 64 and 92 min respectively.

## Conclusion

This study contributes data towards the determination of the value of isothermal amplification as a diagnostic tool for CDI. Both isothermal methods are easy to use, deliver reliable results with a rapid turn-around time and have a high sensitivity compared to other molecular methods. A high negative predictive value of 98 % was demonstrated for both assays, which provides a rapid reliable result to clinicians treating patients with suspected CDI and diarrhoea enabling a de-escalation of empiric therapy. For laboratories dealing with a small number of CDI-requests the option of a single step random access diagnostic method in terms of personnel efficiency is probably cost effective. Both tests are useful for the detection of *C. difficile* with up to 10 specimens per run.

## Abbreviations

BGM: Buffalo Green Monkey
CDI: *Clostridium difficile* infection
CCNA: cytotoxic cell neutralisation assay
EIA: enzyme immunoassay
HDA: helicase-dependant amplification
LAMP: loop-mediated isothermal DNA amplification
MALDI-ToF: Matrix-assisted-laser desorption/ionisation-time of flight
NAAT: nucleic acid amplification test
PaLoc: pathogenicity locus
TC: toxigenic culture
